# Non-paying partnerships and its association with HIV risk behavior, program exposure and service utilization among female sex workers in India

**DOI:** 10.1186/1471-2458-14-248

**Published:** 2014-03-13

**Authors:** Sandra Mary Travasso, Bidhubhusan Mahapatra, Niranjan Saggurti, Suneeta Krishnan

**Affiliations:** 1 Division of Epidemiology, St John’s Research Institute, St John’s National Academy of Health Sciences, Bangalore 560034, India; 2 Population Council, 142 Golf Links, New Delhi 110003, India; 3 Women’s Global Health Imperative, RTI International, 351 California Street, Suite 500, San Francisco, CA, USA

**Keywords:** Female sex workers, Non-paying partners, HIV prevention resources, India

## Abstract

**Background:**

In India, HIV prevention programs have focused on female sex workers’ (FSWs’) sexual practices vis-à-vis commercial partners leading to important gains in HIV prevention. However, it has become apparent that further progress is contingent on a better understanding of FSWs’ sexual risks in the context of their relationships with non-paying partners. In this paper, we explored the association between FSWs’ non-paying partner status, including cohabitation and HIV risk behaviors, program exposure and utilization of program services.

**Methods:**

We used data from the cross-sectional Integrated Behavioral and Biological Assessment (IBBA) survey (2009–2010) conducted among 8,107 FSWs in three high priority states of India- Maharashtra, Andhra Pradesh and Tamil Nadu. Multiple logistic regression was used to examine the association between non-paying partner and cohabitation status of FSWs with HIV risk behaviors, program exposure and utilization of program services.

**Results:**

FSWs reporting a non-paying partner were more likely to be exposed to and utilize HIV prevention resources than those who did not have a non-paying partner. Analyses revealed that FSWs reporting a non-cohabiting non-paying partner were more likely to be exposed to HIV prevention programs (adjusted OR: 1.7, 95% CI: 1.3 – 2.1), attend meetings (adjusted OR: 1.5, 95% CI: 1.2 – 1.8), and visit a sexually transmitted infections clinic at least twice in the last six months (adjusted OR: 1.6, 95% CI: 1.3 – 1.9) as compared to those reporting no non-paying partner. That said, FSWs with a non-paying partner rarely used condoms consistently and were more vulnerable to HIV infection because of being street-based (p < 0.001) and in debt (p < 0.001).

**Conclusion:**

FSWs with cohabiting partners were more likely to be exposed to HIV prevention program and utilize services, suggesting that this program was successful in reaching vulnerable groups. However, this subgroup was unlikely to use condoms consistently with their non-paying partners and was more vulnerable, being street based and in debt. The next generation of HIV prevention interventions in India should focus on addressing relationship factors like risk communication and condom negotiation, including specific vulnerabilities like indebtedness and street based solicitation among women in sex work.

## Background

Female sex workers (FSWs) are a key population at risk for HIV infection in India, with a prevalence that is 20 times higher than the general population [1]. Beginning in 2003, large scale, targeted HIV prevention programs, including the Avahan program funded by the Bill & Melinda Gates Foundation, were implemented among FSWs and other key populations in high prevalence states, and resulted in significant increases in the uptake of HIV prevention services and safer sexual behaviors, and reductions in the number of sexually transmitted infections (STIs) [2,3]. According to an UNAIDS report, by the year 2010, India saw a drop of 56% in the incidence of new HIV infections [4]. Despite these important gains in HIV prevention, HIV prevalence continues to be relatively high among FSWs in India at about 4.9% [1]. In some southern states, up to 15% of FSWs are reported to be living with HIV [5]. Further gains in prevention are likely to depend on a more nuanced understanding of the factors contributing to the persistence of high levels of risk among FSWs.

To date, HIV prevention research and programs have mainly focused on FSWs’ sexual practices vis-à-vis commercial partners, and their practices within the context of intimate relationships have been less consistently addressed. It is well known that women in India have limited decision-making agency, especially in the economic and sexual realms, in the context of their intimate relationships [6-9]. A large body of evidence has highlighted the ways in which entrenched gender inequalities compromise women’s access to and use of critical reproductive and sexual health resources [6,9-11]. Yet, relatively few studies have explored FSWs’ sexual risks and their access to HIV prevention services in context of their relationships with non-paying partners [12-15]. Data from targeted interventions in the high prevalence states of southern India from 1995 to 2008 indicate that FSWs’ consistent use of condoms with paying clients significantly increased from 58.6% to 83.7% (p < 0.001) [16], although condom use with non-paying partners remained a low 10% [12]. However, studies have also found that HIV and STI prevalence was lower among married FSWs compared with widowed and unmarried FSWs [17-19].

The available evidence suggests that several factors are likely to shape FSWs’ HIV risks in the context of relationships with non-paying or intimate partners. For example, married FSWs are more likely to solicit clients on the street or at non-brothel-based venues whereas single FSWs are more likely to be brothel- or home-based [17]. When street-based, women may not identify themselves as sex workers, making it difficult to identify and engage with them on HIV prevention and heightening their risk of HIV infection [18]. Of the 80% of FSWs reporting indebtedness, debt has been found to be more common among FSWs who have an intimate partner; indebtedness is also associated with an increased likelihood of experiencing violence and STI symptoms [19]. Moreover, intimate partner violence (IPV) is widely prevalent among FSWs, although prevalence estimates vary due to differences in definition and reference period. In one study in Chennai, nearly all (98%) surveyed FSWs reported IPV [20] while about a third (35%) reported IPV in another study in Goa [21]. FSWs who face violence from clients or non-paying partners are less likely to use condoms consistently even with clients or utilize HIV prevention program services [22]. However, there is limited literature on the influence of these factors on the uptake of HIV prevention services by FSWs reporting a non-paying partner, including those with whom they are cohabiting. The aim of this paper is to explore the association between FSWs’ non-paying partner status, including cohabitation, and their HIV risk behaviors, HIV prevention program exposure, participation in groups/events and health care service utilization in three states in India.

## Methods

### Data

Data for this study are drawn from the Integrated Behavioral and Biological Assessment (IBBA), a cross-sectional survey conducted in 2009–10 among 8,107 FSWs to generate information on HIV risk practices, STI and HIV prevalence, and program exposure in six states of India. The surveys were conducted as part of the Avahan HIV prevention program, which was launched in 2003 and implemented over 5 years [23]. The program focused on key populations including FSWs, their clients and men who have sex with men, in the high HIV prevalence states in India. The main components of the program were peer-led outreach and education, condom promotion and distribution, clinical services for managing STIs, community empowerment and structural interventions.

Cross sectional IBBA surveys were conducted to assess the outcomes and impact of Avahan by tracking trends in STI and HIV prevalence and risk practices and to project future trends among key populations at risk in these regions [24]. Round 1 was conducted between 2005 and 2007 and Round 2 between 2009 and 2010. In this paper we analysed the IBBA Round 2 survey data conducted among FSWs in three high priority states of central and southern India- Maharashtra, Andhra Pradesh and Tamil Nadu. While Maharashtra and Andhra Pradesh were recognized as having high HIV prevalence (5% and higher), Tamil Nadu has been a high priority states among the key groups – FSWs, men who have sex with men, intravenous drug users, migrants and truckers - by the HIV sentinel survey of India in the year 2007 [25]. A probability sampling method was adopted using conventional cluster sampling for brothel-based and home-based sex workers and conventional time-location cluster sampling for street-based FSWs. Sampling weights were used to account for the differential recruitment of FSWs by typology within districts, differential probabilities of selection across districts and differential non response rates. Details on program survey design, district selection, sample size calculation, participant recruitment and evaluation design have been described elsewhere [24,26].

Trained interviewers used a structured survey instrument to gather information on socio-demographic characteristics, sex work history, partner status, duration of relationship, marital and cohabitation status of non-paying partners, condom use, program exposure, participation in groups/events and service utilization. The questionnaire was implemented in the local languages and pre-tested for appropriateness and accuracy of the translations.

### Measures

#### Non-paying partner status

The key independent variable of interest in this study is FSWs’ non-paying partner status. Non-paying partners refer to partners who were involved in sexual relationships with FSWs without paying cash for sex such as husband, boyfriend or live-in partner. FSWs were first categorized into two groups: FSWs who had non-paying partners and FSWs who had no non-paying partners based on a question regarding whether they had a non-paying regular partner at the time of the survey. We further categorized FSWs by their cohabitation status with their non-paying partner. FSWs who had non-paying partners were classified into three groups: cohabiting with husband (“married-living with husband”), cohabiting with a non-paying partner other than husband (“unmarried-living with partner”, “married-living with partner other than husband”, “divorced/separated-living with partner”, “widowed-living with partner”) and not cohabiting with husband/non-paying partner (“unmarried-living alone”, “married-living alone”, “divorced/separated-living alone”, “widowed-living alone”). These three categories were developed to examine the influence of cohabitation status on HIV risk behaviors and HIV prevention services uptake as we believe that the security, social acceptance and autonomy may differ among the FSWs reporting these different partner types. FSWs in these three categories were compared with FSWs who did not have a non-paying partner in terms of the outcomes of interest.

#### Outcome variables

The outcome variables in this study included indicators on condom use, HIV prevention program exposure, participation in groups/events and service utilization. Inconsistent condom use was measured separately for occasional clients (defined as the clients who came to the FSW only once or a few times more but the FSW does not remember their faces or do not know them), regular clients (defined as the clients the FSWs recognize well, who come to the FSW repeatedly and they know these clients) and non-paying partners (husband, boyfriend or live-in partner). Separate questions on frequency of condom use (every time, most of the time, sometime and never) with each type of partners were asked. FSWs who reported using condoms every time were considered consistent condom users (coded as 0) else considered as inconsistent condom users (coded as 1). HIV prevention program exposure was measured in terms of contact by a peer educator in the one month prior to survey. FSWs’ participation in groups/events at the time of this survey was assessed using three measures: membership in a self-help group, membership in a sex worker collective, and attendance at meetings organized by non-governmental organizations (NGOs). Separate questions with dichotomous response categories (yes, no) were asked for these three measures on participation in groups/events. Service utilization was measured in terms of whether FSWs had made at least two visits to an STI clinic in the six months prior to survey. Responses on the measures of program exposure, participation in groups/events and service utilization were re-coded to make them suitable for analysis (no = 0, yes = 1).

#### Vulnerability index

A summary measure of vulnerability was created to understand the degree of vulnerability associated with FSWs’ cohabitation status. Empirical research suggests that soliciting in street based setting, experiencing violence and being in financial debt make FSWs vulnerable to HIV risk [18,19,22]. Therefore, these three factors were considered to create the summary measure of vulnerability. Separate questions were asked for each of these three variables. FSWs who reported their main place of solicitation as either street, railway station, cinema hall, park or any other public places were considered as soliciting in street based settings (coded as 1) else soliciting in non-street based settings (coded as 0). Questions with dichotomized response categories (no, yes) on any experience of violence in the previous six months and being in financial debt at the time of the survey were asked. These three items were added to create the vulnerability index with a maximum score of 3 and minimum score of 0.

#### Socio-demographic and sex work characteristics

The following socio-demographic and sex work related variables were included in the analyses as potential confounders: age (continuous), education (no formal education, have formal education), income other than sex work (no, yes), financial debt (no, yes), residential status (localite, non-localite), place of solicitation (home-based, brothel-based and street based), age at sex work debut (continuous), and client volume in a week (<10, 10+), alcohol consumption in past one month (no, yes), experience of physical violence in past six months (no, yes).

### Statistical analyses

First, we compared the profile of FSWs by partner status, and differences were tested using z-test and t-test as appropriate. Multiple logistic regression models were fitted to examine the association between partner status and the outcome variables (HIV risk practices, program exposure, participation in groups/events and service utilization). Multiple logistic regression models were adjusted for age, education, income other than sex work, financial debt, place of solicitation, residential status, alcohol consumption, experience of violence, age at sex work debut and client volume. Global weights were calculated for the study sample using mapping data collected prior to the survey to estimate the size of the FSW population and district-specific sampling weights. The global weight was a proportion of sampled FSWs for each district against the total number of FSWs in all 3 states. Details of the weighting methodology have been described elsewhere [26]. Adjusted odds ratios (adjusted OR) and their corresponding 95% confidence intervals (CI) were calculated. Statistical analyses were performed using STATA version 12.1.

### Ethics statement

Approvals were obtained from the Health Ministry Screening Committee, Government of India to conduct the survey. The research ethics committees of the Indian Council for Medical Research and FHI 360 approved the survey protocols. Independent community advisory boards and community monitoring boards were formed in each of the sampled districts to provide inputs on the local context, address issues arising during the course of the survey, and strengthen human subjects’ protections. Verbal informed consent was obtained from eligible individuals prior to data collection.

## Results

### FSW characteristics

The profile of FSWs by their non-paying partner status has been presented in Table 1. The mean age of the FSWs was approximately 32 years (±7.3 years). Statistically significant differences were observed in the socio-demographic and other characteristics of FSWs who have a non-paying partner compared to those who did not report a non-paying partner (Table 1). Compared to the proportion of FSWs who did not have a non-paying partner, lesser proportion of FSWs having a non-paying partner had no formal education (56% vs. 52%, p = 0.001), lesser proportion had no sources of income other than sex work (57% vs. 38%, p < 0.001), and greater proportion had financial debt (64% vs. 76%, p < 0.001). Place of solicitation differed between these two groups, with higher percentage of FSWs who had a non-paying partner reported soliciting in street-based settings than their counterparts (75% vs. 68%, p < 0.001).

**Table 1:** Profile of female sex workers according to their status of having non-paying sexual partners in India

**Background characteristics**	**Number of FSWs**	**Have non-paying partner (N = 5235)**	**Have no non-paying partner (N = 2874)**	**Total (N = 8107)**	**P-value**
		**% or mean (SD)**	**% or mean (SD)**	**% or mean (SD)**	
Age (in years), Mean (SD)	8,107	31.6 (7.0)	31.8 (8.1)	31.7 (7.3)	0.194
Had no formal education	4,898	52.2	56.2	53.4	0.001
Had no income other than sex work	4,573	38.3	57.3	43.8	<0.001
Had financial debt	5,271	75.6	64.3	72.3	<0.001
Localite* to the city/village of interview	5,903	72.6	73.5	72.8	0.378
Place of solicitation					
*Home*	1,259	14.8	14.8	14.8	1.000
*Brothel/Lodge/Dhaba*	1,851	10.3	17.7	12.5	<0.001
*Street*	4,997	74.9	67.5	72.7	<0.001
Cohabitation status				71.0	
*Cohabiting husband*	3,792	72.4			
*Cohabiting non-paying partner*	1,066	20.4			
*Non-cohabiting non-paying partner*	377	7.2			
Median duration of relationship (in years)	5,235	11			
Age at sex work debut, Mean (SD)	8,107	25.9 (6.0)	25.5 (6.7)	25.8 (6.2)	0.010
Had sex with 10+ clients in a week	3,458	36.5	36.1	36.4	0.734
Consumed alcohol in past one month	3,563	39.0	41.8	39.8	0.020
Experienced physical violence in past six months	1318	17.3	16.6	17.1	0.448
Vulnerability index^1^, Mean (SD)	8107	1.7 (0.7)	1.5 (0.8)	1.6 (0.8)	<0.001

SD: Standard deviation.

^1^FSWs who were soliciting at street based sites, had financial debt and experienced physical violence were considered to be vulnerable [See Methods].

^*^Localite has been defined in the context of place of residence of FSWs. If the place of residence (city/town/village) and place of interview (city/town/village) is same, then individual was considered localite; otherwise non-localite.

More than two-thirds (71%) reported having a non-paying partner at the time of the interview. Among the 5,235 FSWs reporting a non-paying partner, over two-thirds (72%) noted that the partner was a cohabiting husband; one-fifth (20%) identified a cohabiting partner whom they were not married to and a few (7%) mentioned having a non-cohabiting non-paying partner. The median duration of relationship with non-paying partners was 11 years.

### Association between non-paying partner status and HIV prevention resources

Partner status was significantly associated with program exposure, participation in groups/events and service utilization (Table 2). FSWs having a non-cohabiting non-paying partner had the highest odds of program exposure, attending meetings organized by an NGO, being a part of self-help groups, and visiting an STI clinic at least twice in the last six months compared to those having no non-paying partners. They were followed by FSWs reporting a cohabiting non-paying partner; and then FSWs reporting a cohabiting husband when compared with FSWs reporting no non-paying partner. Nearly nine in ten (87%) FSWs who had non-paying partners reported inconsistent condom use with them. Among those who had non-paying partners, inconsistent condom use was highest with their cohabiting husband (97%), followed by cohabiting non-paying partner (67.4) and lastly with a non-cohabiting, non-paying partner (58.4%). No statistically significant differences were observed in the odds of using condoms with regular clients, except among FSWs reporting a non-cohabiting non-paying partner: the latter group was more likely to use condoms with regular clients than those with no non-paying partner (adjusted OR: 1.4, 95% CI: 1.0-1.8).

**Table 2:** Unadjusted percent and adjusted odds ratio predicting HIV risk behavior and HIV prevention resources uptakewith cohabitation status as the main predictor variable among female sex workers in India

**Dependent variables**	**Cohabitation status**				**Cohabiting husband vs. no non-paying partner**	**Cohabiting non-paying partner vs. no non-paying partner**	**Non-cohabiting non-paying partner vs. no non-paying partner**
	**No non-paying partner**	**Cohabiting husband**	**Cohabiting non-paying partner**	**Non-cohabiting non-paying partner**			
	**N = 2874**	**N = 3792**	**N = 1066**	**N = 377**	**AOR (95% CI)**^**1**^	**AOR (95% CI)**^**1**^	**AOR (95% CI)**^**1**^
**HIV risk behaviors**							
Inconsistent condom use with occasional clients^2^	10.1	11.6	12.3	7.0	1.2 (1.0-1.4)	1.2 (1.0-1.6)	0.7 (0.5-1.0)
Inconsistent condom use with regular clients^3^	11.6	13.2	12.4	14.9	1.1 (1.0-1.4)	1.0 (0.8-1.3)	1.4 (1.0-1.8)^*^
Inconsistent condom use with non-paying partner^4**^	-	96.6	67.4	58.4			
**Program exposure**							
Contacted by a peer educator in past one month	52.2	57.8	60.0	66.8	1.1 (1.0-1.3)^*^	1.2 (1.0-1.4)^*^	1.7 (1.3-2.1)^**^
**Participation in events/groups**							
Member of a self help group	21.3	26.4	29.0	28.7	1.2 (1.1-1.4)^*^	1.3 (1.1-1.6)^*^	1.3 (1.1-1.6)^*^
Member of sex worker collective	34.7	41.9	46.8	39.4	1.2 (1.1-1.4)^**^	1.6 (1.4-1.9)^**^	1.1 (0.9-1.4)
Attended meetings organized by NGO	46.7	53.1	54.9	59.8	1.1 (1.0-1.3)^*^	1.2 (1.0-1.4)	1.5 (1.2-1.8)^**^
**Service utilization**							
Visited an STI clinic at least twice in last six months	46.5	51.8	54.5	60.8	1.1 (1.0-1.2)	1.1 (1.0-1.3)	1.6 (1.3-1.9)^**^

^*^P < 0.05, ^**^P < 0.001.

^1^AOR: Adjusted Odds Ratio; CI: Confidence Interval.

^2^Computed among those had sex with occasional clients, N = 7788.

^3^Computed among those had sex with regular clients, N = 7053.

^4^Percentages representing those who reported having a non-paying partner, N = 5235.

Multiple logistic regression models were adjusted for age, education, income other than sex work, financial debt, place of solicitation, residential status, alcohol consumption, experience of violence, age at sex work debut and client volume with having non-paying partner as the reference category.

### Vulnerability index

**Figure 1:**
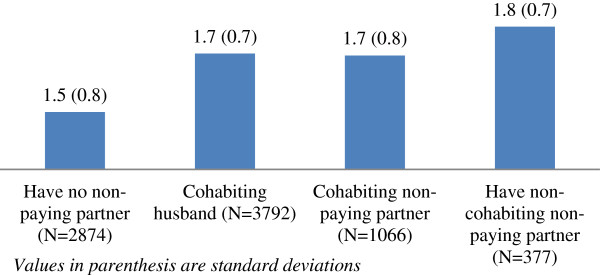
**Mean vulnerability score among female sex workers.** Values in parenthesis are standard deviations.

FSWs who have a non-paying partner had higher vulnerability score compared to those who did not report a non-paying partner (1.7 ± 0.7 vs. 1.5 ± 0.8, p < 0.001) (Figure 1). FSWs reporting a non-cohabiting non-paying partner scored the highest on the vulnerability index (1.8 ± 0.7) followed by FSWs reporting a cohabiting husband (1.7 ± 0.7) and those reporting a cohabiting non-paying partner (1.7 ± 0.8).

## Discussion

In this study we explored the association between FSWs’ non-paying partner status and their HIV risk behaviors, HIV prevention program exposure, participation in groups/events and health care service utilization in three high priority states in India. We observed no differences in the HIV risk behaviors between FSWs reporting a non-paying partner and those with no non-paying partners. However, FSWs with a non-paying partner were more likely to be exposed to HIV prevention program services, participate in groups/events and utilize services compared to those reporting no non-paying partner, despite having higher vulnerability scores. Our analysis shows that there is a strong association between the vulnerability of FSWs and their HIV prevention services uptake indicating that the HIV prevention program was successful in reaching the most vulnerable section of this key population at risk.

Recent research has indicated that even though FSWs with non-paying partners had higher vulnerability, the HIV prevalence among them is 11% as compared to 18% among those with no non-paying partner [27]. This reflects the reach of HIV prevention programs to FSWs with non-paying partner, which is further supported by findings of our study. Our analysis shows that FSWs having a non-cohabiting non-paying partner were most likely to avail HIV prevention services, followed by FSWs reporting a cohabiting non-paying partner and then by those reporting a cohabiting husband. One of the reasons for higher service utilization among FSWs with non-paying partners could be that they may be less mobile as compared to those with non-paying partners. Lesser mobility of FSWs could make them more accessible to HIV prevention programs. However, more research is required to understand the motivation behind FSWs having non-paying partners accessing prevention services. Further investigation of reasons underlying this observation as to what factors contribute to the higher uptake of HIV prevention services despite being more vulnerable – such as differences in perceptions of HIV/STI risk and motivation to stay healthy – should be explored using qualitative research.

While FSWs in non-paying partner relationships avail HIV prevention program services, inconsistent condom use with these partners is almost universal. Besides the risk of HIV infection associated with inconsistent condom use, it could also result in unwanted pregnancies and abortions [28,29]. Data on factors that can help explicate the observed associations such as reasons for condom non-use with non-paying partners as well as the family planning and abortion experiences of FSWs availing HIV prevention services, which would have been useful to explore the sexual and reproductive health concerns of FSWs, were not available in our dataset. A study in central India revealed a 70% likelihood of the unwanted and unintended pregnancies among HIV positive women [28] while a survey conducted among FSWs in Goa, India revealed that while 45% of FSWs did not use any method of contraception, 26% had experienced an abortion [29]. The study also indicated that exposure to HIV prevention programs was not significantly associated with contraceptive use [29].

A possible explanation for the lack of condom use is that FSWs lack condom negotiation skills with non-paying partners. Research from south India found that FSWs whose non-paying partners were aware of their sex work practices were more likely to use condoms consistently [30]. Also consistency of condom use was reduced by 18% per year of increase in the duration of the non-paying partner relationship [30]. A study conducted among sex workers and their non-paying partners in Vietnam pointed out that couples who were comfortable in speaking to each other about HIV risk were also more likely to report more consistent condom use within such relationships, hence emphasizing the need to strengthen communication skills among FSWs in non-paying partner relationships [31].

Community mobilization efforts in India have shown some promise in influencing FSWs’ risks in the context of intimate relationships. For example, the Frontiers Prevention Program conducted in Andhra Pradesh found that after four years of intense community mobilization accompanied by access to HIV prevention services, FSWs were more likely to use condoms with regular partners [32]. In contrast, in Karnataka a state-wide survey among a stratified sample of 1,512 FSWs found that women who are part of a sex-worker collective were more likely to report condom use with paying clients but were no more likely to use condoms with non-commercial sexual partners as compared to those who were not a part of a collective [33].

Future HIV prevention interventions in India should focus on addressing relationship factors such as risk communication and condom negotiation. For example, in South Africa, a randomized community trial of a gender-focused intervention (the Women’s Health CoOp) with FSWs increased their condom negotiation skills which in turn significantly increased condom use with their primary sexual partners at 6-month follow-up (52% vs. 37%, p < 0.05) [34]. Related outcomes included improved communication with the main partner about HIV risk, improved self-efficacy and sexual assertiveness and decreased number of experiences of sexual coercion [34].

Our analysis confirms literature on the vulnerability of FSWs reporting a non-paying partner [18,19]. FSWs reporting a non-cohabiting non-paying partner had the highest vulnerability scores followed by FSWs reporting a cohabiting non-paying partner and a cohabiting husband. Scores were lowest among FSWs having no non-paying partner. Research among FSWs in southern India has found that being in a relationship can exacerbate their indebtedness as they have limited control over financial resources in the context of intimate relationships [19]. Furthermore, FSWs reporting a non-paying partner were more likely to be street-based. Street-based FSWs have been found to be at a significantly higher risk of HIV infection as compared to brothel-based workers because they tend to operate independently and have less access to institutional and social support [18]. The fact that three-fourths of FSWs with a non-paying partner reported being in debt and that a majority of them are engaged in street-based sex work and were older in age (data not shown) points to the need for interventions that address their financial and sex work-related risks. Interestingly, unlike previous studies, we found no significant differences in experience of violence by FSWs’ partner status. Further investigation into FSWs’ perceptions and understanding of violence may shed light on this finding.

The limitations of this study should be noted in interpreting the findings. First, data were self-reported by participants and hence subject to recall and social desirability biases. Second, being cross-sectional data we cannot establish causal relationships between partner status and HIV prevention services uptake, highlighting the need for longitudinal studies. Third, the vulnerability score was created based on only three measures of vulnerability. There are many vulnerability factors among FSWs which could have been incorporated in creating the vulnerability index. However, due to unavailability of additional information in the data set, we were limited with the choice of selection of variable to create the index. The question on debt does not measure extent of indebtedness and the question on violence is subjective. Unfortunately, we are limited by the questions that were posed in the Integrated Behavioural and Biological Assessment (IBBA) survey. Furthermore, experience of violence is typically measured using questions such as in the IBBA and is a limitation of all research on women’s experience of violence. Further research, including qualitative investigations, to consider the nuances of these issues in assessing FSWs vulnerability and its relationship with outcomes are warranted. Despite these limitations, our analyses show a strong association between vulnerability of FSWs and HIV prevention service uptake indicating that the program was able to reach out to the most vulnerable FSWs.

The fourth phase of national AIDS control program in India has been launched with the vision that by the year 2020 there will be a significant reduction in new HIV infections building on the already successful targeted interventions [35]. With declining incidence rates of new HIV infections in India and inconsistent condom use with non-paying partners among FSWs being almost universal, the next generation of HIV prevention interventions among FSWs should emphasize the need for universal condom use in efforts to achieve zero new infections in India. Our findings suggest that strengthening the currently existing HIV prevention efforts should include components focused on enhancing FSWs’ HIV risk communication and condom negotiation in the context of non-paying or intimate relationships. The study findings demonstrate that HIV prevention programs have been able to reach the most vulnerable group which has resulted in a lower HIV prevalence among them, these efforts should continue. One the other hand, HIV prevention programs have not been able to reach the FSWs with no non-paying partner to a great extent. Therefore, HIV prevention programs in India need to put more effort in reaching out and providing services to FSWs with no non-paying partners.

## Conclusion

Our study findings indicate that the HIV prevention program in India was successful in reaching the most vulnerable section of this key population at risk. However, despite significant uptake of large scale HIV prevention program services, a majority of FSWs did not use condoms with non-paying partners. FSWs having non-paying partners remain a vulnerable key population at risk of HIV infection. Our findings suggest that the next generation of HIV prevention interventions in India should focus on addressing relationship factors such as risk communication and condom negotiation as well as specific vulnerabilities such as indebtedness and street based solicitation among women in sex work.

## Competing interests

The authors declare that they have no competing interests.

## Authors’ contributions

ST conceptualized the manuscript, conducted the preliminary data analysis, wrote and finalized the manuscript. BM conducted the data analysis, co-wrote and finalized the manuscript. NS provided guidance on data analysis and reviewed manuscript. SK conceptualized the manuscript, offered guidance on data analysis, co-wrote and finalized the manuscript. All authors read and approved the final manuscript.

## Pre-publication history

The pre-publication history for this paper can be accessed here:

http://www.biomedcentral.com/1471-2458/14/248/prepub
